# Long-term survival in 406 males with breast cancer.

**DOI:** 10.1038/bjc.1985.155

**Published:** 1985-07

**Authors:** H. O. Adami, L. Holmberg, B. Malker, L. Ries

## Abstract

Survival was analyzed during a follow-up period of up to 20 years in 406 (97%) of all 420 males in whom breast cancer was diagnosed in Sweden in 1960-1978. After correction for the expected mortality in the general population, cumulated survival rates (with 95% confidence limits) of 66 (58.7-72.5)% and 52 (42.0-62.1)% at 5 and 10 years respectively were found. These figures and the general pattern of relative survival rates were in close accordance with those noted in a concomitant series of female breast cancer. There was a trend toward slightly improved survival rates during the period of study and the median survival times were 3.9, 4.8 and 7.2 years for patients diagnosed in 1960-64, 1965-69 and 1970-74 respectively. Age at diagnosis was seemingly unrelated to the long-term relative survival. We conclude that, except for a slightly higher mean age at diagnosis in males, there is a striking similarity in the natural history of breast cancer between men and women after initial treatment, with an excess death rate which still persists at long-term observation.


					
Br. J. Cancer (1985), 52, 99-103

Long-term survival in 406 males with breast cancer

H.-O. Adamil, L. Holmberg', B. Malker2 & L. Ries3

1Department of Surgery, University Hospital, Uppsala, Sweden; 2The Cancer Registry, The National Board of
Health and Welfare, Stockholm, Sweden; and 3National Cancer Institute, National Institutes of Health,
Bethesda, USA.

Summary Survival was analyzed during a follow-up period of up to 20 years in 406 (97%) of all 420 males
in whom breast cancer was diagnosed in Sweden in 1960-1978. After correction for the expected mortality in
the general population, cumulated survival rates (with 95% confidence limits) of 66 (58.7-72.5)% and 52
(42.0-62.1)% at 5 and 10 years respectively were found. These figures and the general pattern of relative
survival rates were in close accordance with those noted in a concomitant series of female breast cancer.
There was a trend toward slightly improved survival rates during the period of study and the median survival
times were 3.9, 4.8 and 7.2 years for patients diagnosed in 1960-64, 1965-69 and 1970-74 respectively. Age at
diagnosis was seemingly unrelated to the long-term relative survival. We conclude that, except for a slightly
higher mean age at diagnosis in males, there is a striking similarity in the natural history of breast cancer
between men and women after initial treatment, with an excess death rate which still persists at long-term
observation.

Breast cancer in the male is a rare tumour, a fact
which has hindered establishment of its long-term
prognosis. Thus, the number of reports including a
reasonable number of patients is small (Holleb et
al., 1968; Norris & Taylor, 1969; Ribeiro, 1977;
Morgan, 1979; Carlsson et al., 1981; van Hazel et
al., 1981 (unpublished); The Cancer Registry of
Norway, 1975) and some of them are based on
selected  material  (van  Hazel  et  al.,  1981
(unpublished)).

There is a great need for correction of survival
rates in male breast cancer for deaths due to other
causes in order to permit comparison between
materials with differing age distributions, and with
survival figures for female breast cancer. These
requirements have only been met in a small number
of studies, however, from which corrected 5-year
survival rates of 43% to 60% (Norris & Taylor,
1969; Mausner, 1969; Ribeiro, 1977; Morgan, 1979;
The Cancer Registry of Norway, 1975) and 10-year
survival rates of 26% to 28% (Ribeiro, 1977;
Morgan, 1979) have been reported. A relative
survival rate of 25% at 15 years has been found in
Norway (The Cancer Registry of Norway, 1975).

Breast cancer in the male has traditionally been
considered to carry a worse prognosis than that in
the female (Norris & Taylor, 1969; Peltokallio &
Kalima, 1969; Crichlow et al., 1972a), but this
concept has been contradicted in some recent
communications (Langlands et al., 1976; Morgan,
1979; Hakulinen et al., 1981). The validity of these
comparisons, however, was generally low, because
of the limited number of male patients available for
analysis.

Correspondence: L. Holmberg.
Received 21 December 1984.

The aim of this study was to analyze the long-
term survival in virtually all cases of male breast
cancer diagnosed in Sweden in 1960-1978, with
special reference to temporal trends, late excess
death rate, age as a prognostic factor and possible
differences in prognosis between males and females.
The availability of computerized registers covering
the whole population facilitated a nearly complete
follow-up and permitted acquisition of reliable data
from the general population concerning the
expected mortality.

Materials and methods

The National Swedish Cancer Registry was started
in 1958. Physicians are under obligation to report
all cases of diagnosed cancer to The Cancer
Registry. Furthermore, pathologists and cytologists
separately have to notify every cancer diagnosis
made on surgically removed tissues, biopsies and
cytological specimens, and at autopsies. Hence, the
registry receives reports from both these sources in
95% of the cases. The incompleteness in
registration to The Cancer Registry has been
estimated to be , 5% (Mattsson, 1977).

The cancer file is annually linked to The Causes
of Death Registry and the dates of death and
causes of death are transferred. In addition, a last
date of contact is obtained by linking the registry
data with an updated living registry covering the
total Swedish population. On the other hand, there
has been no attempt to follow up all patients
actively.

For all cases the closing date of this analysis was
December 31, 1979. In the 19-year period from
1960 to 1978, 420 cases of malignant male first

?) The Macmillan Press Ltd., 1985

100      H.O. ADAMI et al.

breast cancers were diagnosed in Sweden. Of these,
6 (1.4%) cases were excluded from the survival
analyses, since the diagnosis was made incidentally
on autopsy. Furthermore, 8 (1.9%) did not have
any follow-up and were excluded from the
calculations. As a result a total of 406 patients,
with the age distribution shown in Table I, were
available for follow-up and included in the
analyses. A review of the original reports to the
Cancer Registry for men aged 45 or less at
diagnosis revealed that in 15 out of 16 cases the
tumour was unequivocally invasive according to the
histopathologic examination.

Table I Age distribution

Age at diagnosis,

years        No.      %
<15           1       0
15-24          1       0
25-34          3       1
35-44         10       3
45-54         48      12
55-64         86      21
65-74        154      38
?75         103      25
Total        406     100

For calculating the survival rates, the actuarial or
life-table method was used (Ederer et al., 1961).
The observed survival rate is the proportion of
persons alive at a specified time after diagnosis. The
relative survival rate is the ratio between the
observed survival in the patient group and the
expected survival for that group if they had the
same mortality as the total Swedish population with
respect to age, sex and calendar time. The relative
survival thus estimates the probability of escaping
the risk of dying from breast cancer.

The expected survival rates were calculated on
the basis of death rates from life tables distributed
by sex, 1-year age groups and 5-year calendar
periods (The Swedish National Central Bureau of
Statistics, 1962-1981). The expected figures are
based on the mortality in the total Swedish
population. No correction was made for breast
cancer mortality which is negligible in the male
population.

The survival measures may have been influenced
by sampling errors. Therefore, the standard error is
given as a measure of the confidence that must be
taken into account when interpreting the results.
The   standard   error  was    computed   from
Greenwood's formula (Greenwood, 1926), assuming
that the expected survival has no variance.

Results

The overall relative 5- and 10-year survival rates for
all patients were 66 and 52% respectively (Table
II). After 10 years of observation, the number of
individuals available for analysis was small and the
estimates uncertain. The survival curve showed a
continuing decline, however, not only of observed
but also of relative survival (Figure 1), indicating a
persistent excess death rate in males with breast
cancer 10 and 15 years after diagnosis.

The relative survival among all 12,319 female
patients with first breast cancers in Sweden in
1959-63, determined in a concomitant study
(Adami et al., 1985), is shown in Figure 1. The
patterns of the survival curves are strikingly similar
and the data do not indicate a more unfavourable
prognosis in males than in females with breast
cancer.

The number of patients was too small to allow
an evaluation of possible significant changes in
relative survival related to year of diagnosis during
the period of registration (Table II). There was a
consistent trend, however, toward improved

100-

80

'o60J

40-
20

0            5            10           15

Time (y)

Figure 1 The cumulated observed (0) and relative
(0) survival with 95% confidence limits in 406 men
with breast cancer diagnosed in Sweden in 1960-1978.
For comparison the RS is given for 12,087 women with
breast cancer diagnosed in Sweden in 1960-1964 (0)
(Adami et al., 1985).

BREAST CANCER AND SURVIVAL IN MALES  101

survival in more recently diagnosed patients (Figure
2). Thus, the 5-year relative survival rates were
56% (42.5-69.0), 62% (48.2-75.9) and 72% (61.1-
82.8) for patients diagnosed in 1960-64, 1965-69
and 1970-74 respectively. The corresponding
median observed survival times were 3.9, 4.8 and
7.2 years.

10 -0

80 -
"7 60

C,)

40-

O  I    ,             ,             ,~I

o            5             lo           15

Time (y)

Figure 2 Cumulated relative survival by period of
diagnosis. (0) 1960-64, n=91; (A) 1965-69, n=88;
(7) 1970-74, n=135; (0) 1975-78, n=92. Male
breast cancer, Swedish Cancer Registry.

There was no tendency suggesting any prognostic
impact of age at diagnosis (Table III; Figure 3).

Discussion

The present results were based on complete follow-
up of virtually all male patients with breast cancer
diagnosed in the whole of Sweden during a 19-year
period. The calculation of relative survival was
based on figures for the expected mortality which
were derived from the same population and
adjusted for changes with time. We therefore
consider the validity of the study as acceptable, and
accordingly regard the results as representative for
the Swedish population. Although the results are
based, as far as we know, on the largest material
published hitherto, the number of patients available
for analysis after more than 10 years was so small
that the possible influence of a sampling error

0-4
0

cn

0l

0O

\0

oo

00 00
0% 00

I oo
00~

00 %

0% N

oo %
oo N

W o-
Om 00

0

0%WI)O n
- N oo
00 le m
oo0 C14 0
ON10 R
oo r- I

00

0%

1-

0%

0%
0%

._4

*tu

0 3

0%

_ )
c, . X

0.

t o

0%);

. o

.0 ;

00
r 0

no)
0._
_" C't

0) W_

. O)

0

O) tf

0_

Cd t

0

i..
0%
. _)

a)
owD

\o
(i

Q
0%
0~

0

tk

cr 00 oo%
0 0   0 a
lo: 0~ r.- -4

(1 o    IT  W _

-    1    1

0 . (N) -

r- mt 0

0 W) - (NI

0T cl C4 ci

0- Nc %10

00     e

0% 1%0 qr-
( %08 VI m t

N   ON% 00
oo tn" --

90 00 %0

- VIf) 0 W)

O- O m

.0
Q

.0 q;LI
0~ 9t

.0
Q

-0
0_

102      H.-O. ADAMI et al.

100

a~~~~~~C (j

00                            0
I~~~~~~~~~~~~~~~~~~~~ I~~~~~~~~~~~~~~~~~~~~~~~~~~~~~

3g ~~~~~e oocoNC                           60                   i     >    ,

e~000  I

e6 60 -

O o                                             0               5              lo0 e
eF-                                           Cl ON r  )T             (

ed;               I I 1 oo                Figure 3  Cumulated relative survival by age a40

n v  4     S    N 4  t 0  X

0%~~~~~~~~~~~

0               5              10

Cd       3       0 t                            a                 o   prevented definiTime (y)

>~~

>E .0  t~      2: 'It ON 0                  Figure 3 Cumulated relative survival by age at

0    PI-                                  diagnosis. (0) 45-54y, n=48; (A) 55-64y, n=86; (7)

t e    i                                    65-74y, n = 154; () ? 75y, n= 103. Male breast

>

=;       u   C   00 xo * ^                cancer, Swedish Cancer Registry.

-v                  ccprevented definite conclusions concerning long-term
* ~ g            0                   eltv    uria adltsurvival from being drawn.

a8-. z:        x   o                      femalThe traditionally held view  that male breast

4   00 'ItTen               cancer carries a poorer prognosis than    breast

cancer in the female is contradicted by the fact that
=   00 u   co               relative survival and late excess death rate in the
ON                                       406 patients reported here do not exceed those of

10 c                    female patients. This view has been explained in

00 Un1-

some studies (Peltokallio & Kalima, 1969; Norris &
Taylor,  1969; Crichlow   1972a, b; Robison   &
o       o                   Montague, 1982) by the assumption      that the

r x- 0               necessarily  more  central location  and  earlier
E.L~                0%re~ invasion of the surrounding tissues in male breast
;  0 .                      cancer implies earlier regional and distant spread.

Our data do not indicate that these anatomic
n  o                    p-. ~  factors lead to a more aggressive clinical course

than in female breast cancer.

A Swedish series of male breast cancer collected
O F                0% ~.c ~ z             in 1968-73 (Carlsson et al. 1981) included 135 of

o csthe patients reported  here. A  comparison  of
3  ~ ~ ~   ~~~i ~~~           presenting symptoms, distribution into stages, and

treatment, with those reported in earlier studies

z~~~

0   -                  ~~~~~~(Mausner 1969; Crichlow et al., 1972b; Langlands

8                            ~~~~~~~~~~et al., 1976; Ribeiro, 1977; Morgan, 1979; Robison

& Montague, 1982) showed that the Swedish
0                      ~~~~~~~~patients had approximately the same clinical picture
F-                             ~~~~~~~~~at presentation and included the same proportion

of cases in stage I. However, there were somewhat

BREAST CANCER AND SURVIVAL IN MALES  103

more patients in stage II, whereas in other series
there have been more stage III and IV patients. As
a consequence, more patients could be treated with
primary surgery in the Swedish study and fewer
patients were treated with radiotherapy alone.
These differences are not large, however, and in the
studies on patients with a somewhat more
unfavourable stage distribution both a long-term
survival similar to ours (Mausner, 1969; Morgan,
1979) as well as a worse prognosis (Crichlow et al.,
1972b; Ribeiro, 1977) have been found.

The tendency for the survival rates to be better
among the male patients diagnosed in the later part
of the study parallels identical observations on
female breast cancer in Sweden (Adami et al.,
unpublished). In females the possibility has been
discussed that better relative survival rates in later
years may have been a result of increased
diagnostic activity. There is no screening or other
specific diagnostic measure directed against cancer
of the male breast.

Another explanation might be that a more
benign subgroup of the disease is increasing in
incidence (Fox, 1979). Delay in diagnosis has also
been pointed out as a factor influencing prognosis
and has been reported to be a common occurrence
in male breast cancer (Mausner, 1969; Peltokallio &

Kalima, 1969; Crichlow, 1972a). The question
whether these two latter factors have changed over
the years was not elucidated in our study. It is thus
difficult to explain the trend toward a more
favourable course. This finding should be
interpreted with reservation, as the numbers are
small and the observation time short for the latest
period.

A higher age at diagnosis does not entail a worse
prognosis, according to our study and several
others in which survival figures corrected for age
have been compared (Morgan, 1969; Mausner,
1969; Peltokallio & Kalima, 1969; The Cancer
Registry of Norway, 1975; Robison & Montague,
1982). The finding contrasts with the regular trend
toward a more favourable course in younger
patients, which was recently established for female
breast cancer in Sweden (Adami et al., 1985).

Except for a slightly higher mean age at
diagnosis in the males, we conclude that there is a
striking similarity in the natural history of breast
cancer between men and women after initial
treatment, with an excess death rate which still
persists at long-term observation.

This study was supported by grants No. 83:256 and
84:143 from the Swedish Cancer Society.

References

ADAMI, H.O., MALKER, B., MEIRIK, O., STONE, B.,

BERGKVIST, L. & PERSSON, I. (1985). Age as a
prognostic factor in breast cancer. Cancer (in press).

THE CANCER REGISTRY OF NORWAY 1953-1967. (1975).

The Norwegian Cancer Society, Oslo.

CARLSSON, G., HAFSTR6M, L. & JONSSON, P.E. (1981).

Male breast cancer. Clin. Oncol., 7, 149.

CRICHLOW, R.W. (1972a). Carcinoma of the male breast.

Surg. Gynecol. Obstet., 134, 1011.

CRICHLOW, R.W., KAPLAN, E.L. & KEARNY, W.H.

(1972b). Male mammary cancer. Ann. Surg., 175, 489.

EDERER, F., ACTELL, L.M. & CUTLER, S.J. (1961). The

relative survival rate: a statistical methodology. Natl
Cancer Inst. Monogr., 101.

FOX, M.S. (1979). On the diagnosis and treatment of

'breast cancer. JAMA, 241, 489.

GREENWOOD, M. (1926). Reports on Public Health and

Medical Subjects, No 33. Appendix 1, The Errors of
Sampling of the Survivorship Tables. HMSO: London.

HAKULINEN, T., PUKKULA, E., HAKAMA, M.,

LEHTONEN, M., SAXEN, E. & TEPPO, L. (1981).
Survival of cancer patients in Finland in 1953-1974.
Ann. Clin. Res. (suppl. 31), 48.

HOLLEB, A.I., FREEMAN, H.P. & FARROW, J.H. (1968).

Cancer of male breast. I. N. Y. State J. Med., 68, 544.

LANGLANDS, A.O., MACLESS, N. & KERR, G.R. (1976).

Carcinoma of the male breast: A report of a series of
88 cases. Clin. Radiol., 27, 21.

MATTSSON, B. (1977). Completeness of registration in the

Swedish Cancer Registry. The National Board of
Health and Welfare 1977. HS 15.

MAUSNER, J.S. (1969). Mammary cancer in Philadelphia.

Cancer, 23, 271.

MORGAN, G.W. (1979). Carcinoma of the male breast in

East Anglia 1960-1976. Clin. Oncol., 5, 331.

NORRIS, H.J. & TAYLOR, H.B. (1969). Carcinoma of the

male breast. Cancer, 23, 1428.

PELTOKALLIO, P. & KALIMA, T.V. (1969). Malignant

tumours of the male breast in Finland. Br. J. Cancer,
23, 480.

RIBEIRO, G.G. (1977). Carcinoma of the male breast. A

review of 200 cases. Br. J. Surg., 64, 381.

ROBISON, R. & MONTAGUE, E.D. (1982). Treatment

results in males with breast cancer. Cancer, 49, 403.

THE SWEDISH NATIONAL CENTRAL BUREAU OF

STATISTICS, OFFICIAL STATISTICS OF SWEDEN:
POPULATION CHANGES. Annual Publ. 1960-1979,
Stockholm 1962-1981.

				


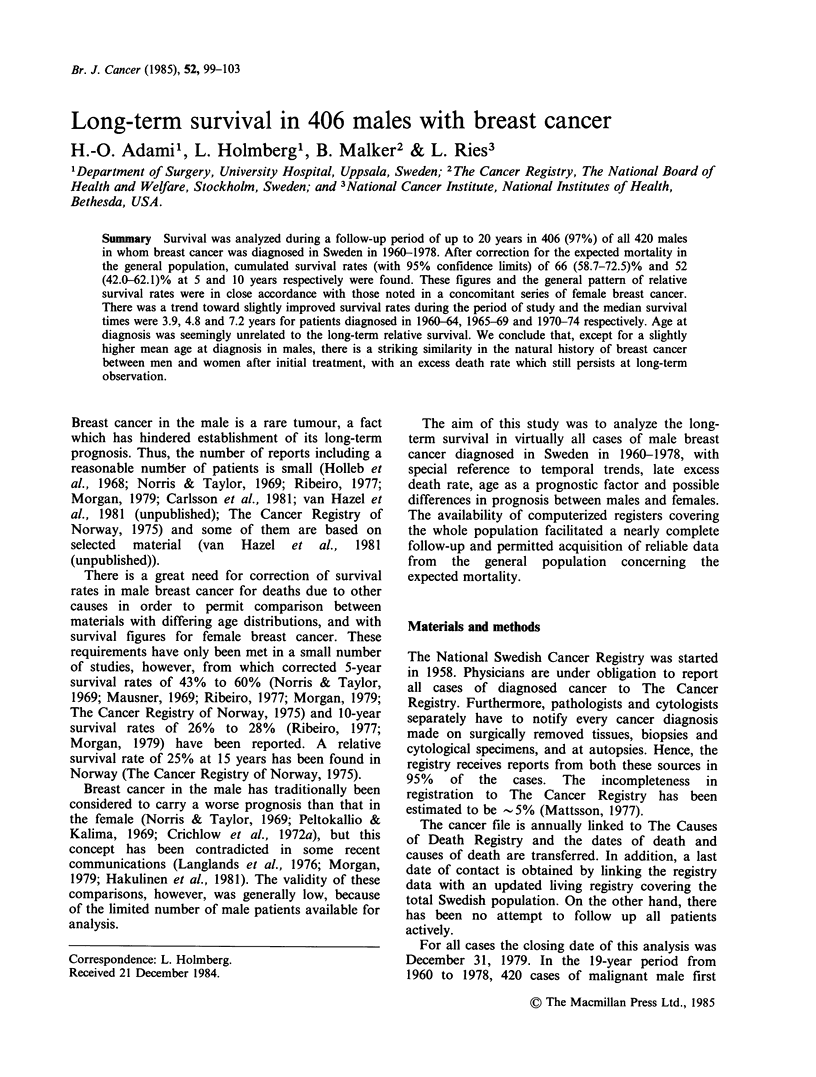

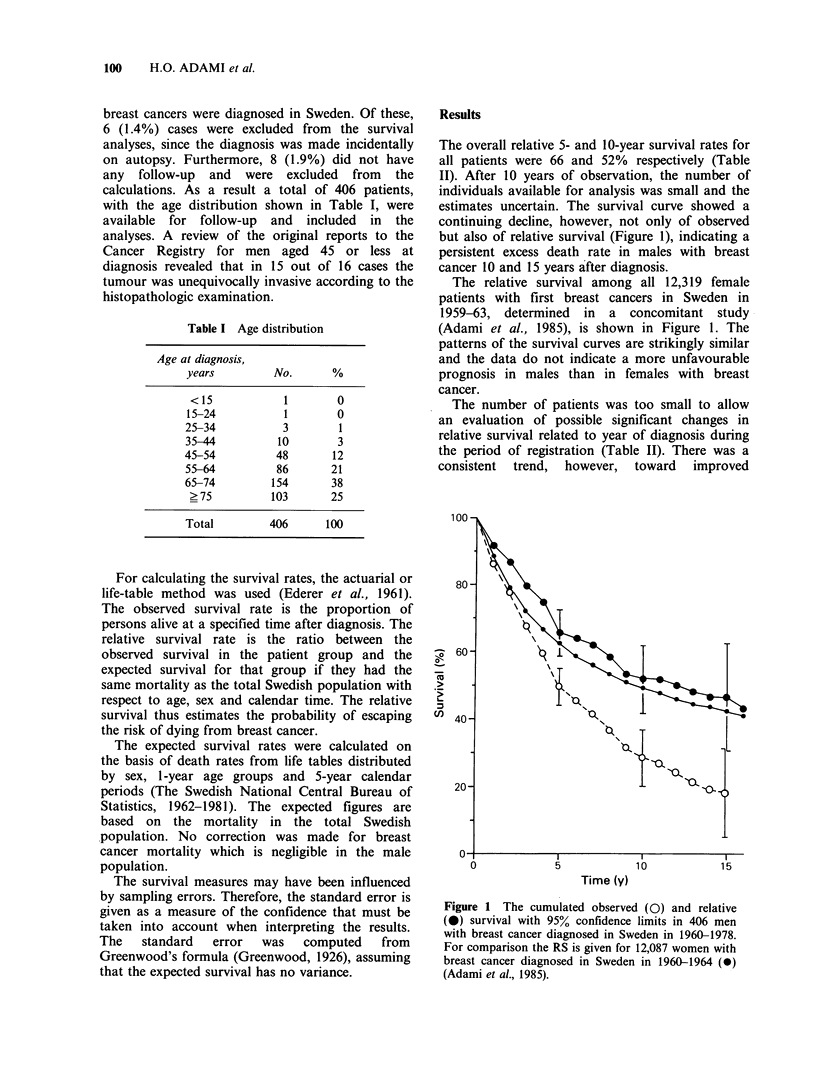

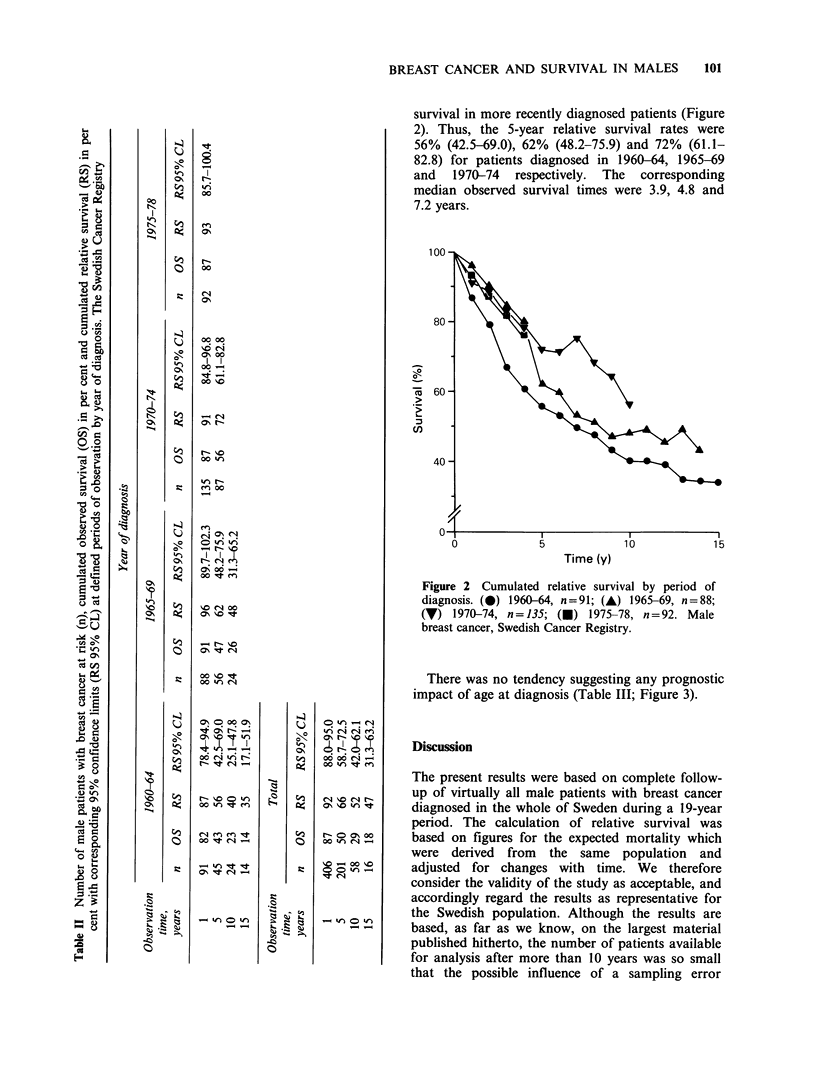

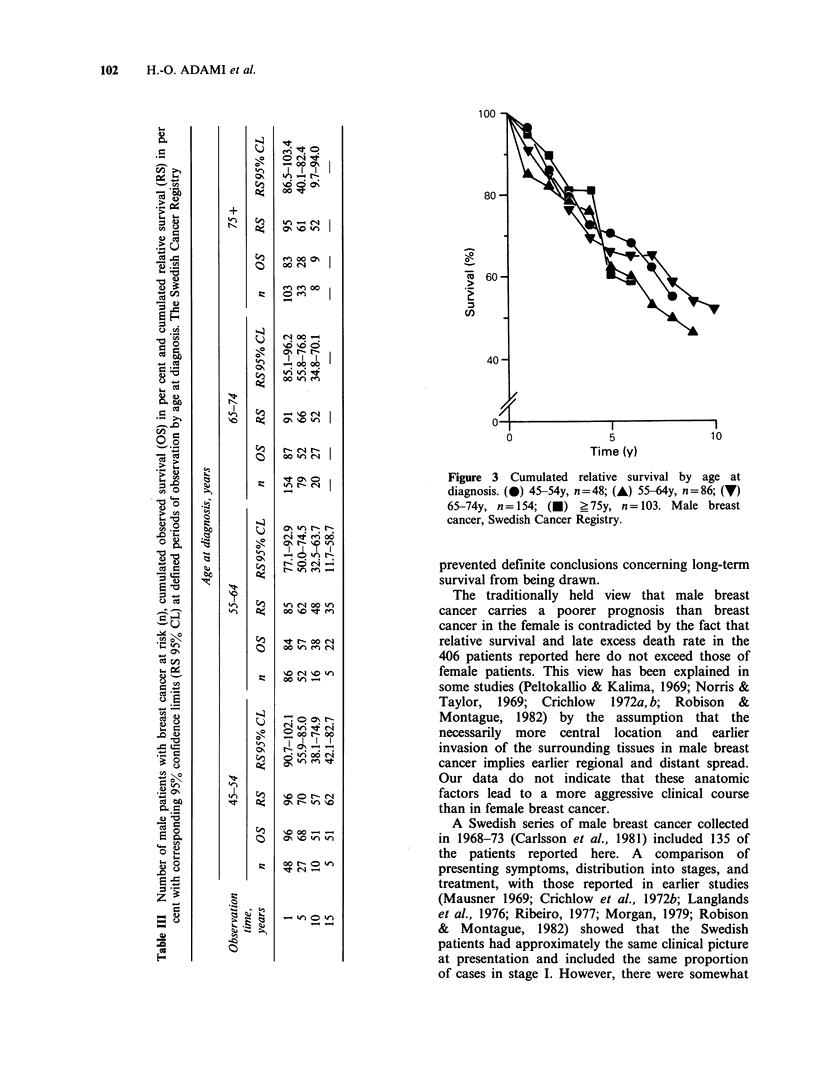

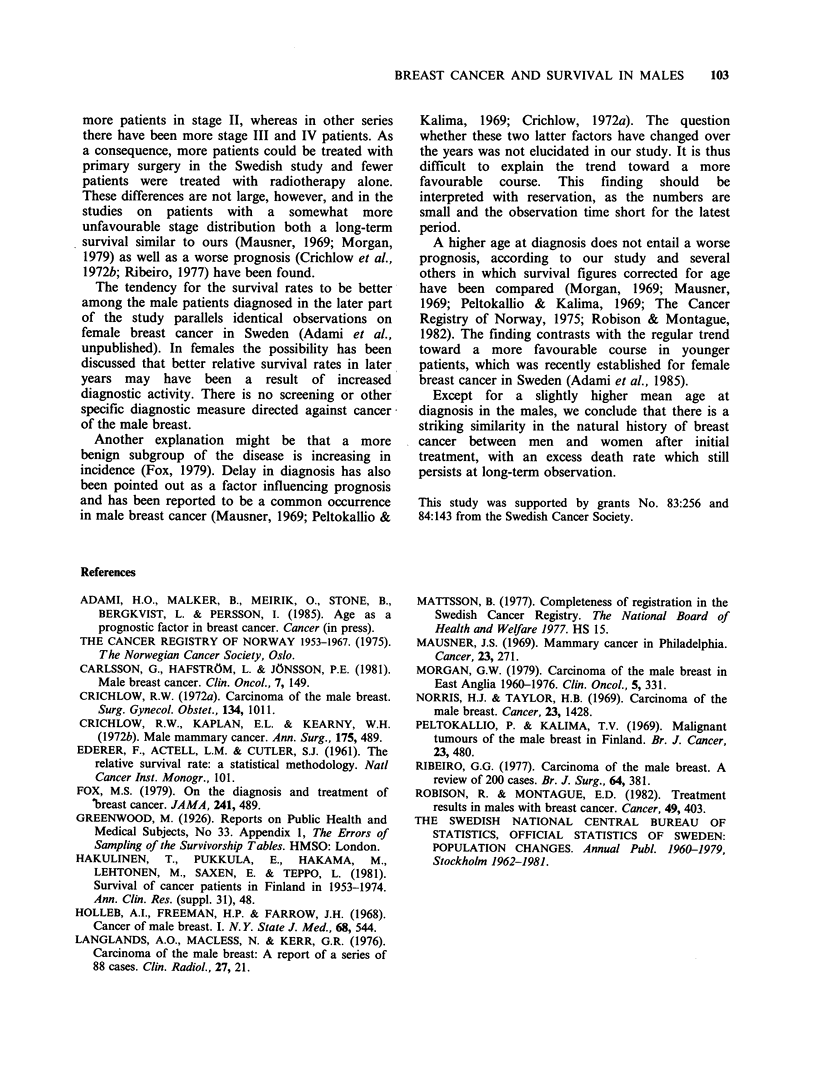

